# Dissociable Neural Mechanisms Underlying the Modulation of Pain and Anxiety? An fMRI Pilot Study

**DOI:** 10.1371/journal.pone.0110654

**Published:** 2014-12-15

**Authors:** Katja Wiech, Robert Edwards, Graham Lorimer Moseley, Chantal Berna, Markus Ploner, Irene Tracey

**Affiliations:** 1 Nuffield Department of Clinical Neurosciences (Nuffield Division Anaesthetics), University of Oxford, John Radcliffe Hospital, Headley Way, OX3 9DU, Oxford, United Kingdom; 2 Oxford Centre for Functional Magnetic Resonance Imaging of the Brain, Nuffield Department of Clinical Neurosciences, University of Oxford, John Radcliffe Hospital, Headley Way, OX3 9DU, Oxford, United Kingdom; 3 Department of Anesthesiology, Perioperative and Pain Medicine, Harvard Medical School, Brigham & Women's Hospital, Chestnut Hill, Massachusetts, United States of America; 4 Sansom Institute for Health Research, University of South Australia, Adelaide 5000, Australia & Neuroscience Research Australia, Sydney, Australia; 5 Department of Psychiatry, University of Oxford, OX3 7JX, Oxford, United Kingdom; 6 Department of Neurology, Technische Universität München, Munich, Germany; National Scientific and Technical Research Council (CONICET), Argentina

## Abstract

The down-regulation of pain through beliefs is commonly discussed as a form of emotion regulation. In line with this interpretation, the analgesic effect has been shown to co-occur with reduced anxiety and increased activity in the ventrolateral prefrontal cortex (VLPFC), which is a key region of emotion regulation. This link between pain and anxiety modulation raises the question whether the two effects are rooted in the same neural mechanism. In this pilot fMRI study, we compared the neural basis of the analgesic and anxiolytic effect of two types of threat modulation: a “behavioral control” paradigm, which involves the ability to terminate a noxious stimulus, and a “safety signaling” paradigm, which involves visual cues that signal the threat (or absence of threat) that a subsequent noxious stimulus might be of unusually high intensity. Analgesia was paralleled by VLPFC activity during behavioral control. Safety signaling engaged elements of the descending pain control system, including the rostral anterior cingulate cortex that showed increased functional connectivity with the periaqueductal gray and VLPFC. Anxiety reduction, in contrast, scaled with dorsolateral prefrontal cortex activation during behavioral control but had no distinct neural signature during safety signaling. Our pilot data therefore suggest that analgesic and anxiolytic effects are instantiated in distinguishable neural mechanisms and differ between distinct stress- and pain-modulatory approaches, supporting the recent notion of multiple pathways subserving top-down modulation of the pain experience. Additional studies in larger cohorts are needed to follow up on these preliminary findings.

## Introduction

Understanding how the brain modulates pain has become a major focus of basic and clinical pain research. In particular, the observation that cognitive processes such as beliefs can attenuate responses to noxious stimulation (e.g., [1], [2]) has buttressed the biopsychosocial model of pain, and lent empirical support to strategies that target altered cognitions as a way to reduce pain [3], [4]. Functional neuroimaging studies have highlighted the role of the ventrolateral prefrontal cortex (VLPFC) in belief-related modulation of pain. Perceived control over pain [5]–[7], religious beliefs [8] and placebo-induced expectancy of reduced pain [9] all result in pain reduction that correlates with increased VLPFC activation [6]. Behavioral data suggest that the analgesic effect of beliefs is achieved via a re-interpretation of aversive events (e.g., pain) as less threatening [10]. Although this process might occur rather unconsciously, it resembles reappraisal (i.e., the volitional change in affective meaning) that has been associated with reduction in anxiety and increased VLPFC activation also in other contexts not related to pain [11].

The close interrelation between the modulation of anxiety and pain raises several questions. First, it is unclear whether the reduction in pain is based on a distinct analgesic mechanism or is simply the “after-effect” of reduced anxiety. Given that anxiety is known to amplify pain [12]–[14], a reduction in anxiety could result in less pain because it revokes the basis for amplified pain processing [15]. In a similar vein, the reduction of pain could reduce anxiety without engaging an active anxiolytic mechanism.

Second, anxiety reduction can be accomplished using a variety of behavioral approaches; whether different forms of anxiety-reducing interventions operate via common or distinct neural mechanisms is an open question [16], [17]. For example, perceived control over pain reduces pain as well as anxiety [6]. However, lower anxiety (and pain) levels might also result from the relative absence of threat, brought about by external signals [12], [18]–[20]. The expectation of low-intensity compared to high-intensity stimulation, for instance, is accompanied by lower anxiety and pain ratings for physically identical stimuli [12], [20]. To date, differing types of modulation have only been studied separately, rendering it difficult to directly relate their efficacy and underlying neural mechanisms. A study in rats revealed that providing either behavioral control over a stressful shock (i.e., rats could prevent shock administration by performing certain actions) or a safety cue indicating that no shock was forthcoming reduced anxiety behaviors, but they did so via distinct neural pathways [16].

Here, we used functional magnetic resonance imaging (fMRI) to compare the neural basis of the modulatory effects of “behavioral control” and “safety signaling” (we adopt these terms from [16], and utilize them throughout the manuscript) in the same sample of healthy volunteers. To allow for the investigation of interactive effects between both types of modulation, we employed a repeated measures full-factorial design, combining behavioral control and safety signaling within subjects. We hypothesize that both, control over the noxious stimulation and safety signaling lead to lower ratings of the intensity of pain, perceived threat and helplessness but that their underlying neural mechanisms will be different. Based on previous studies, we hypothesize that the analgesic effect of perceived control involves the ventrolateral prefrontal cortex (VLPFC), whereas external threat engages top-down pathways including the rostral anterior cingulate cortex (rACC) and PAG.

## Materials and Methods

### Subjects

Twelve healthy right-handed individuals (seven females, mean age, 29.9±4.4) participated in the experiment. The subjects were pre-assessed to exclude those with a previous history of neurological or psychiatric illness including chronic pain.

### Ethics statement

All subjects gave written informed consent and the study was approved by the Oxfordshire Clinical Research Ethics Committee.

### Experimental design and protocol

**Figure 1:**
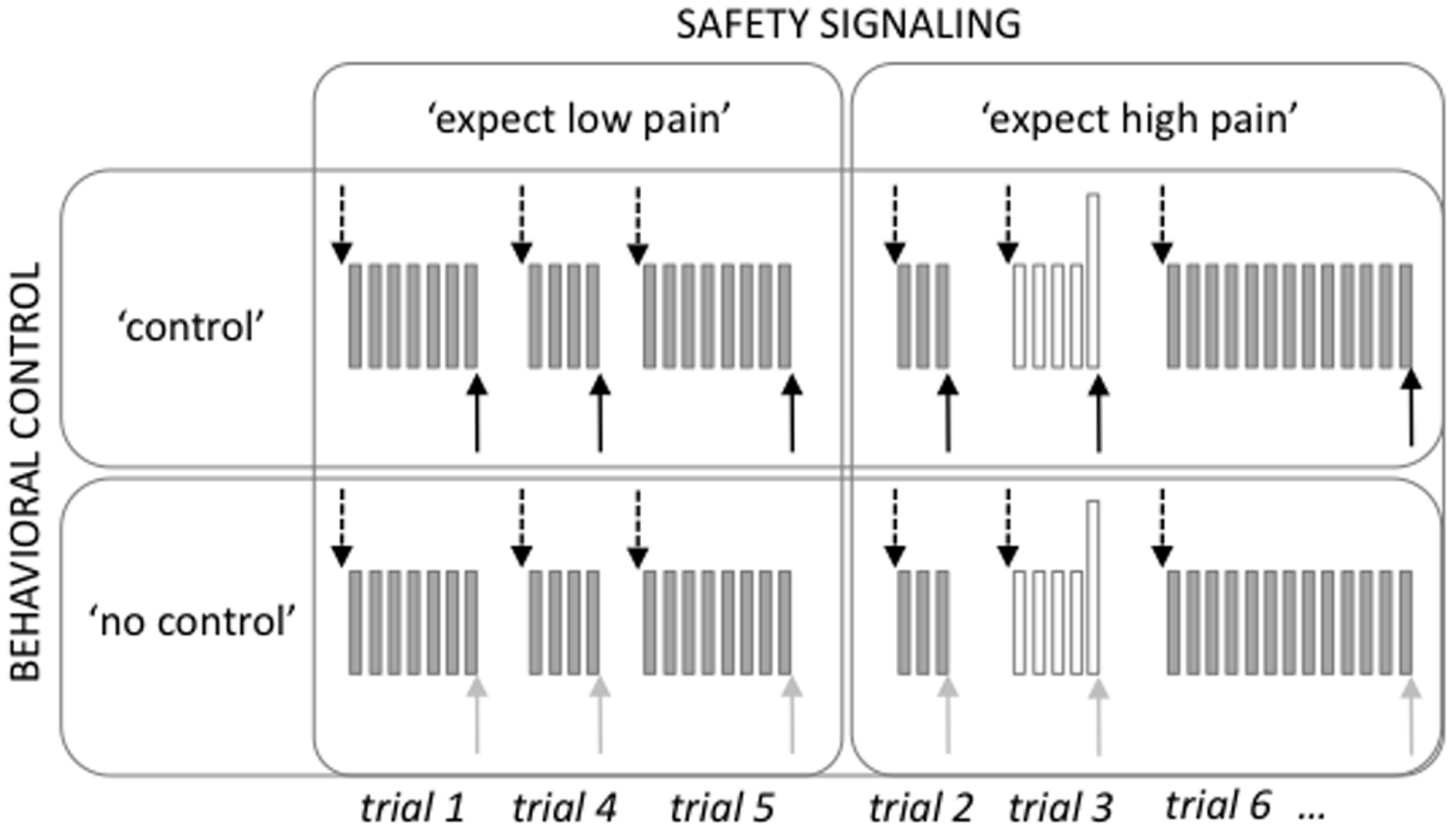
Study design. We used a 2×2 factorial design with the factors BEHAVIORAL CONTROL modulation (i.e., ‘control’ vs. ‘no control’ over the noxious stimulation) and SAFETY SIGNALING modulation (i.e., ‘expect low pain’ vs. ‘expect high pain’ noxious stimulation). During two of the four runs, the noxious stimulation could be stopped by the participant (‘control’). In the other two runs, participants had no control over the stimulation (‘no control’). Prior to each trial, participants were informed by a visual cue whether the upcoming stimulation would only consist of moderately painful stimuli (‘expect low pain’) or could also include high-intensity pain stimuli (‘expect high pain’; arrows pointing downwards). Trials during which high-intensity stimulation was delivered were excluded from the analysis (shown in white). Each trial was followed by ratings of pain intensity, helplessness and threat and a baseline period (not shown). Note that the same number of stimuli was applied in the ‘control’ and the ‘no control’ runs. Arrows pointing upwards indicate button presses that stopped the noxious stimulation in the ‘control’ runs and were performed after the stimulation had stopped in the ‘no control’ runs.

In this study, we used a 2×2 factorial design with the two within-subject factors BEHAVIORAL CONTROL modulation (‘control’ vs. ‘no control’) and SAFETY SIGNALING modulation (‘expect low pain’ vs. ‘expect high pain’; Fig. 1) resulting in four conditions. In the two ‘control’ conditions, subjects were able to stop a repetitive electrical stimulation that was applied to the back of their left hand (see below for details of the stimulation) by pressing a button. In the ‘no control’ conditions the number of stimuli that were applied was predefined and the subjects were told that they had no influence on the stimulation. In fact, using a stimulus-matching paradigm described previously [6], see also below), participants received the same number of stimuli in the ‘control’ and ‘no control’ trials. For the two safety signaling conditions, electrical stimuli delivered to the hand were either all of the same moderately painful intensity (‘expect low pain’) or varied in intensity with rare, single, high intensity stimulus occurring within a train of moderately painful stimuli (‘expect high pain’). In order to differentiate between anxiolytic and analgesic effects of the two forms of cognitive modulation, participants rated the subjective level of both pain and anxiety responses (differentiated into ‘threat’ and ‘helplessness’) after each trial.

The experiment was divided into four runs with thirteen trials per run (six ‘expect low pain’ trials, seven ‘expect high pain trials’). The factor BEHAVIORAL CONTROL was presented in a blocked fashion. Half of the participants started with a ‘control’ run, followed by a ‘no control’ run, the second ‘control’ and the second ‘no control’ run (order: ABAB). The other half of the subjects followed an inverted order (i.e., BABA). The order was alternated between subjects. Subjects were informed via the intercom whether the following run would be controllable or uncontrollable.

During both ‘control’ and ‘no control’ runs, ‘expect low pain’ and ‘expect high pain’ trials were presented in a randomized order. Six seconds prior to the onset of stimulation, subjects were presented with a visual cue. For half of the subjects, a yellow circle signaled an ‘expect low pain’ trial and a blue square signaled an ‘expect high pain’ trial. For the other half of the subjects, these cues were reversed. The cues were randomly assigned to one of the two levels of threat (i.e., yellow circle signaled ‘expect low pain’ in half of the subjects and ‘expect high pain’ in the other half), but remained the same throughout the experiment. The cues were presented in the center of the screen until the end of the subsequent noxious stimulation. During the application of the stimuli, subjects were instructed to visually fixate on the cue, which was replaced by a white cross when the electrical stimulation stopped. In order to control for motor responses that occurred in the ‘control’ runs subjects also had to press a button at the end of the ‘no control’ stimulus trains. They were cued by an enlargement of the fixation cross to press the button as quickly as possible. The white cross enlarged immediately after the noxious stimulation had stopped.

### Stimulation

Prior to the first run, individual current levels were determined and were adjusted between runs if necessary (see Experimental protocol). The mean stimulation intensity across runs was 1.35 mA (SD = 0.05; run 1: M = 1.21, SD = 0.52; run 2: M = 1.31, SD = 0.53; M = 1.41, SD = 0.52; run 4: 1.48, SD = 0.53). The increase in stimulation intensity from run 1 to run 4 that was required to induce comparable intensity levels of pain did not reach statistical significance (as revealed by a repeated measures ANOVA testing one within-subject factor STIMULATION INTENSITY with four levels (run 1–4): F(3,33) = 3.85; p = 0.069). The stimuli were applied to the same site on the back of the left hand throughout the experiment using a commercial electric stimulation device (Digitimer, Constant Current Stimulator, Model DS7A), which delivered trains of 200 µs monopolar square waveform pulses via a silver chloride electrode. For the ‘expect low pain’ trials the interstimulus interval was 500 ms. In three of the ‘expect high pain’ trials, series of ten stimuli with an interstimulus interval of 20 ms occurred intermixed with the regular 500 ms interval stimulation. This high-frequency stimulation induced a strong but tolerable pain, which was confirmed as such for each subject at the end of the calibration procedure. Because we aimed to apply the identical stimulation during ‘control’ and ‘no control’ runs, the number of stimuli per trial delivered in the ‘no control’ runs per trial was determined by the number of stimuli participants had chosen during the previous ‘control’ run [6]. For instance, if a subject had stopped the stimulation after the tenth stimulus in the first trial and after the thirteenth stimulus in the second trial of a ‘control’ run he received ten and thirteen stimuli during the following ‘no control’ run. Note that the order of stimuli was randomized (e.g., the ten stimuli could be applied before or after the thirteen stimuli) to ensure that the subjects did not become aware of the matching procedure. If the first run was uncontrollable, the number of stimuli to apply was matched to the practice run that was performed prior to the four experimental runs. During the second ‘no control’ run, the number of stimuli was matched to the preceding ‘control’ run as described. On average, participants stopped the stimulation after 15 stimuli in both the ‘expect low pain’ and ‘expect high pain’ condition (‘expect low pain’: SD = 9.86; min./max.: 5/37 stimuli; ‘expect high pain’: SD = 10.35; min./max.: 6/39 stimuli). Critically, the number of stimuli applied in the ‘expect low pain’ and ‘expect high pain’ conditions did not differ significantly (t(11) = 0.37, p = 0.722).

### Subjective ratings of pain, threat and helplessness

At the end of each trial subjects rated (i) the mean pain intensity, (ii) the mean threat and (iii) the mean helplessness during the previous trial. Reviews of the broader literature on emotion and stress highlight the centrality of internal perceptions of one's capacities (e.g., ability to control a stressor) and external perceptions of the degree of threat posed by the stimulus or stressor in question [21]. Our factor ‘behavioral control’ was intended to address the former whereas the factor ‘safety signaling’ was introduced to investigate the latter. In order to be able to differentiate the perception of the two aspects, participants were instructed to rate threat and helplessness separately. All ratings were given via a pointer that could be moved in both directions along a Numerical Rating Scale (NRS), which was displayed on the screen. The pain NRS was anchored at left with “0” “not painful at all” and at right with “100”, “strongest imaginable pain”. The threat NRS was anchored with “not threatening at all” and “highly threatening” and the helplessness NRS was anchored with “not helpless at all” and “very helpless”. Participants were given seven seconds for each of the three ratings.

### Experimental protocol

Upon arrival, subjects were provided with written task instructions and gave their informed consent. Subjects were then brought to the MR control room where they were familiarized with the instructions that would be displayed on the computer screen during the experiment, and with the rating procedure.

Before the subjects were positioned in the MR scanner the individual stimulation levels were determined within the scanner room. In order to find an individual level for electrical stimulation, trains of ten 200 µs stimuli of increasing intensities were applied. After each train the subject gave a verbal intensity rating between 0 and 100 using the same NRS they would subsequently complete manually in the scanner. The calibration procedure stopped when participants rated the intensity as 70 or higher. The intensity of the last electrical stimulus was used for the moderately painful stimuli during the experiment. To account for sensitization or habituation to the stimulus, the stimulus intensity was readjusted prior to each run using the same procedure. Prior to the four experimental runs and after the subject had been positioned in the scanner a short practice run was performed to ensure that the participants had learned the association between the visual cue and the subsequent stimulation.

### Image acquisition

MR scanning was performed on a 3T MRI system (Oxford Magnet Technology, Oxford, UK) with the use of a Nova Medical quadrature birdcage coil (Nova Medical, Wilmington, USA). For the functional measurement, 33 axial slices (slice thickness 3 mm, 1 mm gap) were acquired using a gradient echo echo-planar (EPI) T2*-sensitive sequence (repetition time, 2.38 s; echo time, 30 ms; flip angle, 90°; matrix, 64×64; field of view, 192×192 mm^2^). The first four images were discarded to compensate for T1 saturation effects.

### Data analysis

For the trial-by-trial ratings of pain intensity, threat and helplessness an average score for each of the four conditions was calculated for each subject. Mean scores for the three ratings were subjected to repeated measures ANOVAs with the within-subject factors BEHAVIORAL CONTROL (‘control’ vs. ‘no control’) and SAFETY SIGNALING (‘expect low pain’ vs. ‘expect high pain’). Significant ANOVA results were followed-up by post-hoc t-tests (2-tailed). In order to test whether the effect of the two types of modulation on pain was related to the effect on threat and helplessness, bivariate Pearson correlation coefficients (2-tailed test) were calculated for the difference scores (i.e., ‘control’ minus ‘no control’) in pain intensity and threat or helplessness, separately for the behavioral control and safety signaling modulations. Likewise, we tested for similarities between the effect on threat and helplessness by correlating their difference scores between the ‘control’ and the ‘no control’ conditions (Pearsson correlation coefficient; 2-tailed test). Results of these correlation analyses were corrected for multiple comparisons. As explained in the Results section, ratings for threat and helplessness were highly correlated. We therefore decided to use a composite score (i.e., the average of both ratings) as a general measure of anxiety in the regression analyses of the neuroimaging data (see below).

Image processing and statistical analyses were performed using SPM5 (http://www.fil.ion.ucl.ac.uk/spm). Volumes from all runs were realigned to the first volume, unwarped, spatially normalized to a standard echo-planar imaging template included in the SPM software package [22], and smoothed with an isotropic 8mm full-width-at-half-maximum Gaussian filter to account for anatomical differences between subjects and to allow for statistical inference at the group level.

We estimated subject-specific (first-level) general linear models that included regressors for the combination of the two experimental factors (i.e., ‘expect low pain, control’; ‘expect high pain, control’; ‘expect low pain, no control’; ‘expect high pain, no control’; onset at time-point of stimulus delivery) and for the six-seconds time period in which the visual cue was presented. Trials in which a high intensity stimulus was applied were excluded from the analysis to ensure that potential condition differences could not be explained by a difference in noxious input (resulting in twelve trials per run). The four stimulation conditions were modeled according to the duration of the stimulation, which varied depending on the time-point at which the stimuli were stopped, either by the program (‘no control’) or by the participant (‘control’). Serial autocorrelation was modeled as a first-order autoregressive model, and the data were high-pass filtered at a cutoff of 128 s. Statistical inferences were made at the second (between-subject) level by entering the appropriate contrast into an ANOVA.

In a first step, main effects of both factors (i.e., BEHAVIORAL CONTROL modulation: (‘control’–‘no control’) and (‘no control’–‘control’) and SAFETY SIGNALING modulation: (‘expect low pain’–‘ expect high pain’) and (‘expect high pain’–‘ expect low pain’)) and their interactions ([(‘control’–‘no control’)_‘expect high pain’_ – (‘control’–‘no control’)_‘expect low pain’_] and [(‘control’–‘no control’)_‘expect low pain’_ – (‘control’–‘no control’)_‘expect high pain’_] were calculated.

Next, we performed a series of second-level simple regression analyses to identify brain regions whose activation scaled with the reduction of (i) pain or (ii) anxiety (as a composite of ‘helplessness’ and ‘threat’) within the behavioral control modulation and those in which activation scaled with the reduction of (iii) pain or (iv) anxiety (as a composite of ‘helplessness’ and ‘threat’) within the safety signaling modulation. To this end, differential contrasts (e.g., (‘control’ – ‘no control’)) for each participant were regressed against the mean individual behavioral effect (e.g., pain intensity_‘no control’_ – pain intensity_‘control’_). In the following, the difference in pain intensity between the ‘no control’ and the ‘control’ condition or the ‘expect low pain’ and ‘expect high pain’ condition will be referred to as the *analgesic effect* of the behavioral control or safety signalling modulation. Likewise, the difference in the composite score on anxiety will be referred to as the *anxiolytic effect*. We tested for a positive correlation between activation in the right VLPFC and pain reduction through perceived control, as has previously been shown [6]. To this end, a small volume correction (SVC) was applied to the VLPFC using a sphere with 4 mm radius, centred around the reported coordinate of the peak voxel (x,y,z: 36,48,15). Parameter estimates were extracted from the contrast ‘control – no control’ from the same region of interest (peak x,y,z: 39,48,15; 4 mm sphere) and regressed against the difference in pain intensity between ‘control’ and ‘no control’ trials (i.e., pain_‘no control’_ – pain_‘control’_).

Finally, we investigated the functional connectivity of those brain regions showing a positive correlation in one of the simple regression analyses using psychophysiological interaction analyses (PPI; [23]). These PPI analyses were limited to brain regions that have previously been discussed as potential sources of pain and anxiety modulation, namely the prefrontal cortex and rostral anterior cingulate cortex. Our analyses therefore focused on (i) the right VLPFC (x,y,z: 36,48,15), scaling with the analgesic effect of the behavioral control modulation, (ii) the left DLPFC (x,y,z: -24,54,15), scaling with the anxiolytic effect of the behavioral control modulation and (iii) the rostral anterior cingulate cortex (rACC; x,y,z: 15,45,6) and (iv) left DMPFC/DLPFC (x,y,z: -15,27,45), both scaling with the analgesic effect of the safety signaling modulation. In each of the four analyses, a new statistical model was created for each subject in which the PPI regressor was computed as the element-by-element product of the mean-corrected activity in the region of interest given above (defined as a 4 mm sphere around the peak voxel) and a vector coding for the relevant differential effect (i.e., ‘control’ – ‘no control’ for effects of behavioral control modulation and ‘expect low pain – expect high pain’ for effects of safety signaling modulation). The individual contrast images reflecting the interaction between the psychological variable (i.e, ‘control’ – ‘no control’ or ‘expect low pain – expect high pain’) and the activation time course of the region of interest were subsequently entered into a second-level one-sample *t*-test.

A global threshold was set at p<0.001 uncorrected with a minimum cluster extent of five contiguous voxels. Due to the relatively small size of the brainstem, activations in this structure are reported without a minimum cluster extent. All reported coordinates are given in MNI space.

## Results

### Behavioral data

**Figure 2:**
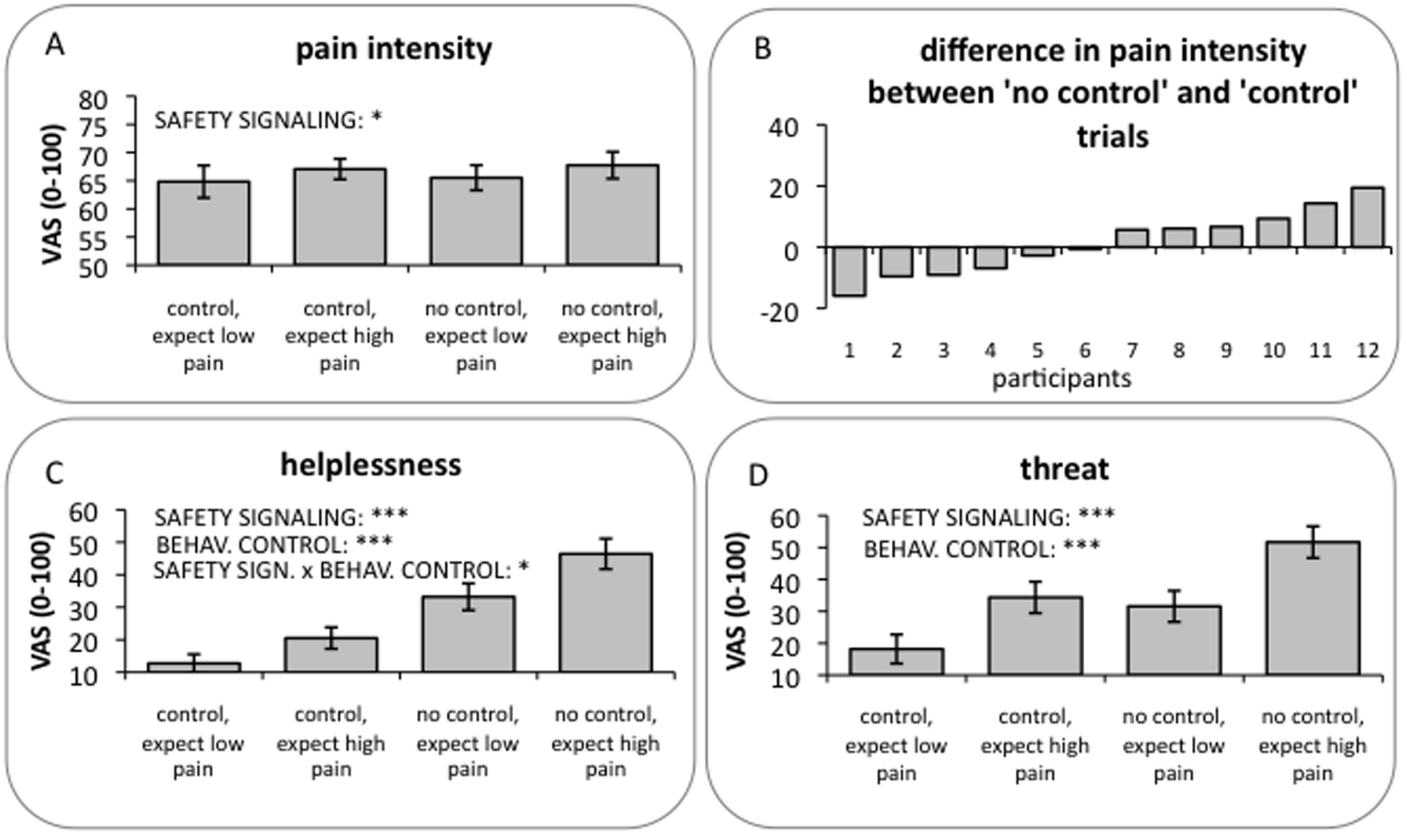
Behavioral results. (A) On average, only the safety signaling modulation (i.e., ‘expect low pain’ relative to ‘expect high pain’ stimulation) showed an analgesic effect. No significant difference in pain ratings was found between ‘control’ and ‘no control’ runs. (B) Individual pain intensity ratings, which show a spread such that half of the participants rated the pain as less intense when they could control it and the other half reported the opposite effect, i.e., increased pain during the two ‘control’ runs. In contrast to the pain intensity ratings, participants felt more helpless (C) and threatened (D) during the ‘expect high pain’ stimulation than they did during the ‘expect low pain’ stimulation and during ‘no control’ runs than they did during the ‘control’ runs. The interaction between both factors only reached statistical significance for the helplessness ratings. Error bars indicate the standard error of the mean. *p<0.05, ***p<0.001.

We first investigated whether the two modulatory approaches had an effect on the intensity of pain as well as on perceived threat and helplessness. As shown in Fig. 2A, participants reported slightly higher pain levels in the ‘expect high pain’ than the ‘expect low pain’ trials (main effect of SAFETY SIGNALING: (F(1,11) = 5.82, p = 0.034). Note that trials in which high-level pain stimuli were applied were excluded from the analysis to ensure that the comparison between conditions was based on physically identical stimuli. Controllability, in contrast, had no effect on pain (no main effect of BEHAVIORAL CONTROL: (F(1,11) = 0.20, p = 0.661) and showed no significant interaction with SAFETY SIGNALING (F(1,11)<0.001, p = 0.997). The analysis of individual data, however, showed that half of the subjects reported less pain when they were able to stop the noxious stimulation whereas the other half reported less pain when the stimulation was uncontrollable (Fig. 2B). Because of these individual differences in the behavioral effects of controllability, no overall difference was observed between pain ratings of controllable stimuli and pain ratings of uncontrollable stimuli.

As expected, participants felt more helpless during the ‘no control’ than the ‘control’ trials (main effect of BEHAVIORAL CONTROL: F(1,11) = 58.48, p<0.001; Fig. 2C), but also during ‘expect high pain’ compared to ‘expect low pain’ trials (main effect of SAFETY SIGNALING: (F(1,11) = 38.80, p<0.001). Furthermore, both factors had an interactive effect on perceived helplessness (F(1,11) = 6.86, p = 0.02). Post-hoc tests revealed that the difference in helplessness between ‘no control’ and ‘control’ trials was more pronounced during the ‘expect high pain’ compared to the ‘expect low pain’ conditions (t(11) = −2.62, p = 0.024).

The analysis of the threat ratings revealed a significant result for both main effects with higher ratings for the ‘expect high pain’ and the ‘no control’ trials (SAFETY SIGNALING main effect: F(1,11) = 42.81, p<0.001; BEHAVIORAL CONTROL main effect: F(1,11) = 34.83, p<0.001; Fig. 2D), but no significant interaction (F(1,11) = 3.34, p = 0.10).

A direct comparison of the two modulatory strategies revealed no significant difference in the reduction of pain (BEHAVIORAL CONTROL: M = 1.38, SD = 10.63; SAFETY SIGNALING: M = 4.45, SD = 6.39; t(11) = 0.94; p = 0.47) or threat (BEHAVIORAL CONTROL: M = 32.94, SD = 19.33; SAFETY SIGNALING: M = 36.92, SD = 19.55; t(11) = 0.68; p = 0.51), suggesting that both types of modulation were equally effective with respect to changes in pain and perceived threat. However, as expected, participants experienced a larger reduction of helplessness as a function of actual control over the electrical stimulation than when they expected low relative to high pain (BEHAVIORAL CONTROL modulation: M = 49.53, SD = 22.44; SAFETY SIGNALING modulation: M = 24.19, SD = 13.45; t(11) = 3.77; p = 0.003).

The correlation analyses revealed that the effect of BEHAVIORAL CONTROL modulation (i.e., ‘control’ – ‘no control’) on threat and helplessness were significantly positively correlated (r = 0.81, p = 0.003). Likewise, the effect of SAFETY SIGNALING modulation (i.e., ‘expect low pain’ – ‘expect high pain’) on threat and helplessness was similarly positively correlated (r = 0.78, p = 0.003), suggesting that at the behavioral level, ratings of threat and helplessness captured a similar experience. We therefore decided to pool across both ratings for a global measure of anxiety for the analysis of neuroimaging data (see Regression analyses with behavioral effects).

Interestingly, no significant correlation was found between the effect of BEHAVIORAL CONTROL modulation on threat or helplessness and its effect on pain (pain and helplessness: r = −0.1, p = 0.76; pain and threat: r = 0.14, p = 0.66). Similarly, the effect of SAFETY SIGNALING modulation on threat and helplessness was unrelated to the effect on pain (pain and helplessness; r = −0.42; p = 0.18; pain and threat: r = −0.51, p = 0.09). Taken together, these results indicate that during both types of modulation, individual differences in anxiety reduction are not necessarily related to individual differences in pain reduction.

### Neuroimaging results

#### Main effect: behavioral control

At the group random effects level, we found a significantly greater increase in the right VLPFC during ‘control’ than during ‘no control’ trials (x,y,z: 36,51,15, z = 3.8, p<0.05, SVC corrected), confirming our previous finding [6]. Additional activation was found in the primary motor cortex and premotor cortex (x,y,z = −33,−21,57; cluster extent: 1408 voxels; z = 5.98), cerebellum (x,y,z = 18, −51, −27; cluster extent: 1929 voxels; z = 5.60), DLPFC (x,y,z = 27, 54, 30; cluster extent: 347 voxels; z = 4.70) and inferior parietal lobe (x,y,z = 39, −48, 45; cluster extent: 353 voxels; z = 4.67). The reverse contrast (i.e., ‘no control’ – ‘control’) revealed significant activation in the right posterior insula (x,y,z = 39,−18,21; cluster extent: 13 voxels; z = 3.64) and contralateral primary somatosensory and motor cortex (SI/MI; x,y,z = 39,−24, 57; cluster extent: 7 voxels; z = 3.40), confirming previous findings of an increased activation in sensory-discriminative brain regions during uncontrollable pain [5].

#### Main effect: safety signaling

A comparison of ‘expect low pain’ versus ‘expect high pain’ trials (i.e., ‘safe – dangerous’) revealed significant activation in bilateral caudate nucleus (right: x,y,z, 12,24,6; cluster extent: 26 voxels; z = 4.02; left: x,y,z = −3,18,6; cluster extent: 6 voxels; z = 3.58). The reverse contrast testing for increased activation during ‘expect high pain’ relative to ‘expect low pain’ trials revealed a significant result only for the cerebellum (x,y,z = −6, −45, −39; cluster extent: 6 voxels; z = 3.50).

#### Interaction between behavioral control and safety signaling

Both interaction analyses revealed no significant results.

In a series of regression analyses we identified brain regions that showed a positive correlation either with the analgesic effect or the anxiolytic effect of either modulation, suggesting that they might be a mediator or source of the effect.

#### Regression analysis on the analgesic effect of behavioral control

**Figure 3:**
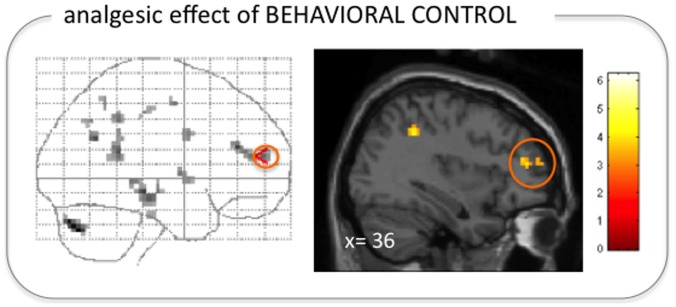
Right VLPFC correlated with the analgesic effect of the behavioral control modulation. The signal level in the rVLPFC during ‘control’ trials as compared to ‘no control’ trials was positively correlated with the difference in pain ratings between those conditions (for display purposes thresholded at p<0.005 uncorrected, minimum cluster extent: 5 voxels; shown on a glass brain on the left and overlaid on a standard structural image (in MNI space) on the right).

As described above, perceived control over the noxious stimulation led to increased pain in some participants while it had an analgesic effect in others (Fig. 2B). However, as expected, the degree of analgesia was accompanied by increased activation in the right VLPFC (x,y,z: 36,45,15; z = 3.01, p<0.05, SVC corrected; Fig. 3), including negative difference scores for those who did not benefit from control over the noxious stimulation. The whole brain analysis revealed additional activation in the left cerebellum (x,y,z: −36,−72,−36; cluster extent: 15 voxels; z = 3.90).

#### Regression analysis on the analgesic effect of safety signaling

**Figure 4:**
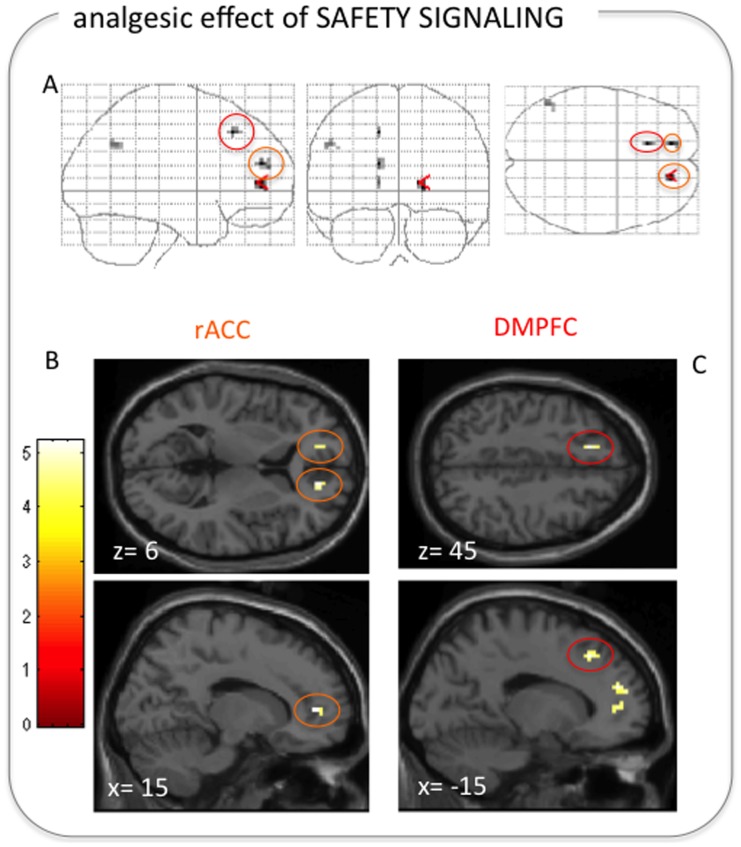
Bilateral rACC and left DMPFC/DLPFC correlated with the analgesic effect of the safety signaling modulation. (A) Brain regions showing a positive correlation with lower pain during ‘expect low pain’ trials than during ‘expect high pain’ trials, displayed on a glass brain (left: sagittal, mid: coronal, right: axial plane; p<0.001 uncorrected, minimum cluster extent: 5 voxels). The bilateral rACC is highlighted in orange. The left DMPFC/DLPFC is highlighted in red. (B) Bilateral rACC and (C) left DMPFC/DLPFC activation overlaid on a standard structural image (in MNI space).

Lower pain intensity ratings during ‘expect low pain’ trials than in ‘expect high pain’ trials were paralleled by increased activation in the rostral anterior cingulate cortex (right rACC x,y,z: 15,45,6; cluster extent: 10 voxels; z = 3.51; left rACC x,y,z: −15,48,6; cluster extent: 5 voxels; z = 3.29; Fig. 4), left DMPFC/DLPFC (x,y,z: −15,27,45; cluster extent: 5 voxels; z = 3.53) and left inferior parietal lobe (x,y,z: −51,−60,36; cluster extent: 10 voxels; z = 3.24).

#### Regression analysis on the anxiolytic effect of behavioral control

**Figure 5:**
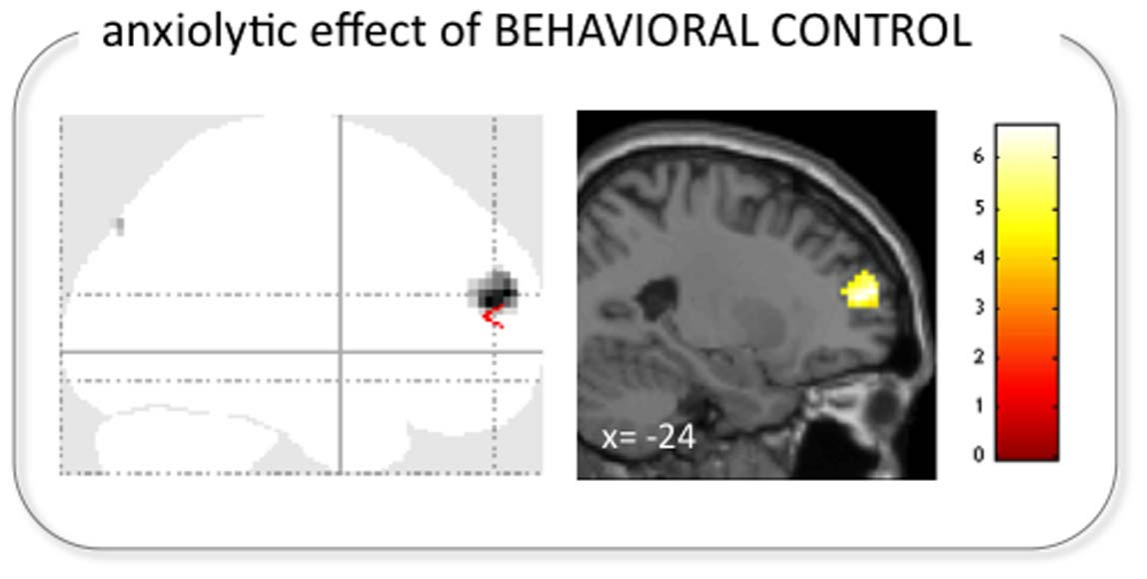
Left DLPFC correlated with the anxiolytic effect of the behavioral control modulation. Activation in the left DLPFC was related to relatively lower ratings of anxiety (i.e., the composite score of perceived threat and helplessness) during the control modulation (p<0.001 uncorrected, minimum cluster extent: 5 voxels; shown on a glass brain on the left and overlaid on a standard structural image (in MNI space) on the right).

Reduction in anxiety through perceived control was accompanied by increased activation in the left DLPFC (x,y,z: −24, 51, 15; cluster extent: 72 voxels; z = 4.02; Fig. 5) and right cuneus (x,y,z: 6,−81,42; cluster extent: 5 voxels; z = 3.34).

#### Regression analysis on the anxiolytic effect of safety signaling

Regression analyses testing for activation that co-varied with the reduction of anxiety during the ’expect low pain’ trials did not reveal any significant results.

In a next step we investigated the functional connectivity of the neural sources of the analgesic and anxiolytic effects using PPI analyses.

#### Functional connectivity of brain regions involved in the analgesic effect of behavioral control

Activity in the right VLPFC that was correlated with pain reduction during ’control’ trials did not show significantly increased functional coupling with any other brain region.

#### Functional connectivity of brain regions involved in the analgesic effect of safety signaling

**Figure 6:**
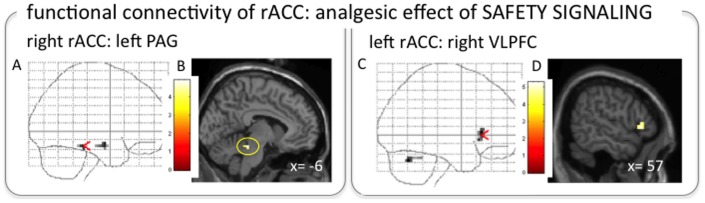
PAG and VLPFC showing increased functional connectivity with the rACC during safety signaling modulation. The right rACC that was correlated with the analgesic effect of the ’expect low pain’ modulation exhibited increased functional connectivity with the left PAG (x,y,z = −6,−33,−18), shown on a glass brain (A), and overlaid on a standard structural image in MNI space (B) (for display purposes thresholded at p<0.005 uncorrected, minimum cluster extent: 5 voxels). The left rACC showed increased connectivity with the right VLPFC (x,y,z = 57,24,0), displayed on a glass brain (C) and overlaid on a standard structural image in MNI space (D) (p<0.001; minimum cluster extent: 5 contiguous voxels).

As shown in Fig. 6, the right rACC correlating with the analgesic effect of safety signaling showed increased functional coupling with the periaqueductal gray (PAG; x,y,z: −6,−33,−18; cluster extent: 2 voxels; z = 3.51), and the right temporal lobe (x,y,z: 54,−6,−15; cluster extent: 3 voxels; z = 3.25) during ‘expect low pain’ compared to ‘expect high pain’ trials. The left rACC, in contrast, exhibited increased functional connectivity with the left VLPFC (x,y,z = 57,24,0; cluster extent: 21 voxels; z = 3.60), extending into the OFC (x,y,z = 51,21,−6; z = 3.49) and with the left cerebellum (x,y,z = −30,−63,−30; cluster extent: 14 voxels; z = 3.66). The left DMPFC/DLPFC as the second region correlating with pain reduction showed increased functional connectivity with the left midbrain reticular formation (x,y,z = −12,−27,−21; cluster extent: 7 voxels; z = 3.83).

#### Functional connectivity of brain regions involved in anxiolytic effect of behavioral control

The left DLPFC that showed a positive correlation in activation with the reduction in anxiety during control trials did not significantly increase its connectivity to other brain regions.

## Discussion

The aim of the present study was to compare the neural mechanisms underlying the analgesic and the anxiolytic effect of two types of modulation of pain responses: One based on perceived personal control over pain and another based on different expectations regarding the stimulus intensity. Our data show that the analgesic and anxiolytic effect can occur independently and that their underlying neural processes differ depending on the type of modulation. While activation patterns found during the behavioral control modulation strongly resembled those known from emotion regulation (i.e., increased activation in the VLPFC for reduction of pain and in the DLPFC for reduction of anxiety), the analgesic effect of the safety signaling modulation engaged the well-known descending pain inhibitory system including the rACC and PAG as well as the VLPFC. Reduction in anxiety during safety signaling had no distinct neural signature.

### Analgesic effect of the two modulatory approaches

In contrast to previous studies [5], [6], [24], perceived control over pain had no effect on the average pain rating of the sample (Fig. 2A), despite the fact that it reduced feelings of threat and helplessness (Fig. 2C, D). As revealed by the analysis of the individual data (Fig. 2B), half of the participants did benefit from control but the reverse effect (i.e., an increase in pain intensity) was observed in the other half. Although perceived control has been shown to be negatively correlated with pain in the past [25]–[27], there is also evidence suggesting that the effects of controllability on pain may vary as a function of subject characteristics [28]. In a study by Rokke and colleagues (which is not perfectly analogous to the present work, but which serves to outline a general principle), subjects were exposed to painful stimuli under conditions in which they either had control, or had no control over their use of pain-coping strategies during the application of noxious stimulation. Decreases in pain in the “control” relative to the “no control” condition were only observed in subjects who scored highly on a measure of self-efficacy for managing pain. That is, the pain-reducing effects of increasing the controllability of a painful situation were selective, and specific to a particular subgroup of participants. In line with this finding, the impact of the controllability of impending pain on central nervous system processing has been shown to vary considerably between individuals [29].

Our finding of greater right VLPFC activation during controllable stimuli than during uncontrollable stimuli replicates previous observations on perceived control [6], [7]. As in our previous study [6], the signal level in the right VLPFC was positively correlated with the degree of pain reduction observed when participants could control the painful stimuli (Fig. 3), which further supports a direct relationship between VLPFC activation and pain reduction. In accordance with our finding, it has recently been reported that patients suffering from functional pain disorders such as Irritable Bowel Syndrome (IBS) or functional dyspepsia show compromised engagement of the VLPFC [30]–[32] and reduced grey matter density in this brain region [33]. Interestingly, the present study did not reveal an increased functional connectivity of the VLPFC during pain relief, suggesting that the change in pain perception was primarily, if not solely driven by a prefrontal mechanism.

The analgesic effect of the safety signaling modulation (i.e., ‘expect low pain’ as compared to ‘expect high pain’ stimulation), in contrast, was related to activation in the rACC (Fig. 4). The right rACC showed increased functional coupling with the PAG (Fig. 6 A, B) – a pattern that is strikingly similar to findings on placebo analgesia [13], [34], [35] and attentional modulation of pain [36]–[38]. rACC and PAG are part of the descending pain control system that modulates spinal transmission of nociceptive information predominantly via opioidergic transmission [39]. Interestingly, the left rACC exhibited increased functional connectivity with the right VLPFC during ’expect low pain’ trials (Fig 6C, D), resembling findings in the context of placebo analgesia [40]. Our findings therefore suggest that neural underpinnings of the experience of pain as being relatively “safe”, or low threat, are not characterized purely by the absence of mechanisms that aggravate pain but rather that this experience involves active down-regulation of pain perception, either via an established cortical-brainstem-spinal circuit or via prefrontal cortex involvement.

### Anxiolytic effect of behavioral control and safety signaling

Similarly to the analgesic effect, the mechanisms that imparted reduced anxiety were different between distinct modulatory approaches. During perceived control, the decrease in anxiety was positively correlated with activation in the left DLPFC (Fig. 5). The DLPFC has been associated with emotion regulation [41]–[46] as has the VLPFC, although the nature of the association is slightly different. Ventral aspects of the lateral prefrontal cortex have been involved in “first-order” executive processes, including the strategic regulation of information [47]–[49]. In contrast, the DLPFC is thought to govern and direct top-down processes of cognitive control including those of the VLPFC [11]. A recent transcranial magnetic stimulation study that manipulated perceived controllability of noxious stimulation yielded findings that strongly support the effects observed in this study [50]. Providing subjects with apparent control over whether or not painful stimuli were administered produced reductions in affective pain ratings. Repetitive TMS of the left DLPFC interfered with the effect while having no influence on the modulation of pain intensity.

Notably, as for the VLPFC, we found no increase in functional connectivity for the left DLPFC during behavioral control. This observation deviates from previous findings that showed an increased connectivity between lateral prefrontal regions including the DLPFC and the amygdala during emotion regulation [51], [52]. Although many reasons might account for this divergence, it should be pointed out that emotion regulation studies commonly use external stimuli such as pictures of fearful faces to induce an emotional state. Such external stimuli are clearly less complex and of less personal relevance than perceived lack of control over an aversive stimulation. Furthermore, participants are often explicitly instructed to employ a regulation strategy whereas in the present study, emotion regulation was assumed to occur spontaneously, as a consequence of perceived control.

Surprisingly, no specific neural correlate could be identified for the anxiolytic effect associated with safety signaling. Although it cannot be ruled out that the absence of evidence relates to a lack of sensitivity of our paradigm, this suggests that the lower threat and helplessness ratings in the ‘expect low pain’ condition are not based on an active down-regulatory neural process but might be the result of a relative lack of threat.

### Limitations of the study

Several aspects of this study might limit the conclusions drawn from it. First, the relatively small sample size only allows for preliminary conclusions. As shown in the glass brain inserts to each figure, activations reported here were, however, very focal. We therefore believe that despite the relatively low number of participants our results provide valuable first insight into the dissociation of pain and anxiety modulation on the neural level, which could inspire follow-up studies in a larger cohort. Second, although perceived control has been shown to modulate pain in some studies, it did not yield a robust effect in the present study. Some participants showed a slight analgesic effect, whereas others reported higher pain intensities during the controllable condition. Because both effects were rather small, it could be argued that perceived control did not lead to relevant levels of analgesia. While the observation that perceived control might rather be a “double-edged” sword that benefits some and while amplifying pain intensity for others might pose a clinical problem (because encouraging perceived control might inadvertently lead to more pain among particular individuals), it is of limited relevance for the present study that did not aim at investigating the potential of perceived control to reduce pain but rather was designed to identify brain regions involved in the modulation of pain through perceived control as an example of internally-based pain modulation. Third, the effects of our two modulatory approaches might not necessarily be conceptually orthogonal. Participants may have felt some control when expecting pain to be lower, and likewise when controlling the length of the stimulation they may have expected pain to be less aversive. It can, however, be stressed that despite this overlap in effect, modulations were triggered by dissociable processes. While participants were aware that the physical properties of the stimulation would be identical in the ‘control’ and the ‘no control’ condition, they expected (and received) different nociceptive input in the two safety signaling (‘expect low pain’ vs. ‘expect high pain’) conditions. We therefore believe that although effects of both manipulations might overlap, the origins of the resulting modulation differ.

### Conclusions

**Figure 7:**
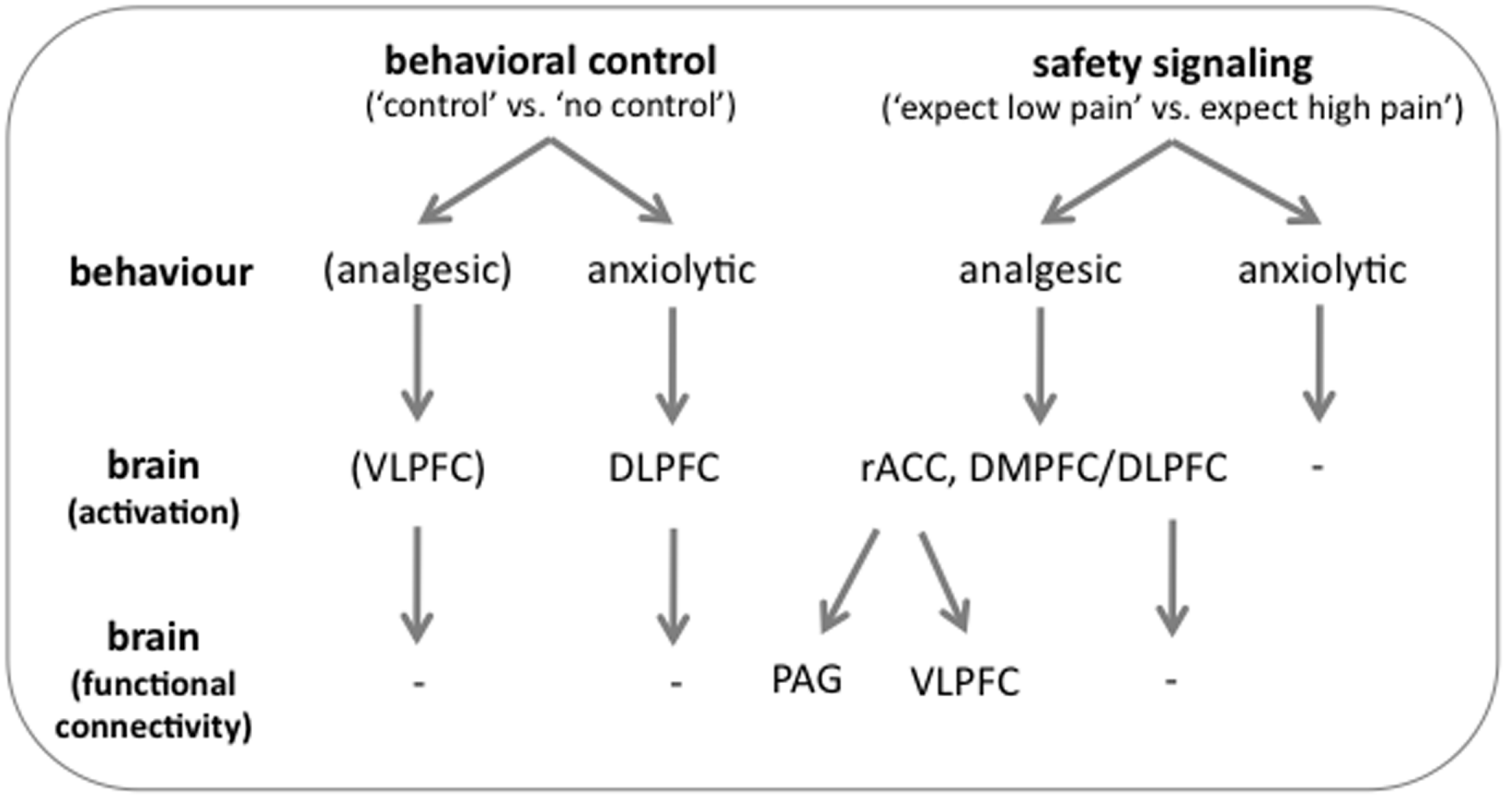
Schematic representation of the neural mechanisms underlying the analgesic and anxiolytic effects of both modulatory approaches. Pain reduction following the ‘control’ modulation was associated with increased activation in the VLPFC. The safety signaling modulation, in contrast, led to pain reduction through the engagement of the descending pain inhibitory system including the rACC, which showed increased functional connectivity with the PAG and the VLPFC. Decreased anxiety during behavioral control was related to increased activity in the DLPFC. No distinct activation could be found for the effect of safety signaling on anxiety.

In conclusion, the data of our pilot study suggest that on the neural level, pain modulation is not simply a by-product of anxiety modulation, but that the two are based on distinguishable neural mechanisms (Fig. 7). While the reduction of pain was associated with activation in the right VLPFC during a control-based form of pain modulation and the engagement of the descending pain inhibitory pathway and right VLPFC during safety signaling, the reduction of anxiety was related to left prefrontal activation. Our finding of a prefrontal pathway to control-based pain modulation, that does not involve the classical descending pain–inhibitory pathway is in line with recent findings [53]. Whether these are indeed two distinct mechanisms or part of a continuum of mechanisms as has been proposed for emotion regulation [54] has to be investigated in future studies. The VLPFC engagement during safety signaling modulation and its increased functional connectivity with the rACC (Fig. 6B, C) clearly point towards a continuum. Moreover, additional research is needed to elucidate the neurochemical processes underlying both types of modulation. In particular, it has to be investigated whether reappraisal also depends on the endogenous opioid system that plays a key role in placebo analgesia [35]. Finally, although the group data showed that both types of modulation were equally effective in reducing pain and anxiety, inter-individual differences exist in the ability to engage the underlying mechanisms. Low ability might hamper successful modulation and should therefore be the target of future studies. The small sample size of our study warrants further investigations in larger cohorts to tease apart neural mechanisms underlying the modulation of pain and anxiety.
